# Protection of Vascular Endothelial Growth Factor to Brain Edema Following Intracerebral Hemorrhage and Its Involved Mechanisms: Effect of Aquaporin-4

**DOI:** 10.1371/journal.pone.0066051

**Published:** 2013-06-21

**Authors:** Heling Chu, Yuping Tang, Qiang Dong

**Affiliations:** 1 Department of Neurology, Huashan Hospital, Fudan University, Shanghai, PR China; 2 Department of Neurology, Huashan Hospital, State Key Laboratory of Medical Neurobiology, Fudan University, Shanghai, PR China; Temple University, United States of America

## Abstract

Vascular endothelial growth factor (VEGF) has protective effects on many neurological diseases. However, whether VEGF acts on brain edema following intracerebral hemorrhage (ICH) is largely unknown. Our previous study has shown aquaporin-4 (AQP4) plays an important role in brain edema elimination following ICH. Meanwhile, there is close relationship between VEGF and AQP4. In this study, we aimed to test effects of VEGF on brain edema following ICH and examine whether they were AQP4 dependent. Recombinant human VEGF165 (rhVEGF165) was injected intracerebroventricularly 1 d after ICH induced by microinjecting autologous whole blood into striatum. We detected perihemotomal AQP4 protein expression, then examined the effects of rhVEGF165 on perihemotomal brain edema at 1 d, 3 d, and 7 d after injection in wild type (AQP4^+/+^) and AQP4 knock-out (AQP4^−/−^) mice. Furthermore, we assessed the possible signal transduction pathways activated by VEGF to regulate AQP4 expression via astrocyte cultures. We found perihemotomal AQP4 protein expression was highly increased by rhVEGF165. RhVEGF165 alleviated perihemotomal brain edema in AQP4^+/+^ mice at each time point, but had no effect on AQP4^−/−^ mice. Perihemotomal EB extravasation was increased by rhVEGF165 in AQP4^−/−^ mice, but not AQP4^+/+^ mice. RhVEGF165 reduced neurological deficits and increased Nissl’s staining cells surrounding hemotoma in both types of mice and these effects were related to AQP4. RhVEGF165 up-regulated phospharylation of C-Jun amino-terminal kinase (p-JNK) and extracellular signal-regulated kinase (p-ERK) and AQP4 protein in cultured astrocytes. The latter was inhibited by JNK and ERK inhibitors. In conclusion, VEGF reduces neurological deficits, brain edema, and neuronal death surrounding hemotoma but has no influence on BBB permeability. These effects are closely related to AQP4 up-regulation, possibly through activating JNK and ERK pathways. The current study may present new insights to treatment of brain edema following ICH.

## Introduction

Vascular endothelial growth factor (VEGF) plays essential roles in the formation of blood vessels during embryogenesis and in many pathological conditions. In recent years, VEGF is considered to have protective effects on many neurological diseases by acting on neurons and glial cells [1]. There are sufficient evidences on its neuroprotective effects in cerebral ischemic models, including apoptosis inhibition and oxidative stress reduction in acute phase as well as neurogenesis promotion and angiogenesis enhancement in chronic phase [2]–[5]. But as to intracerebral hemorrhage (ICH), another type of stroke, few studies have been conducted. It was shown that VEGF and its receptors were up-regulated after ICH, which persisted to 28 d and VEGF had some protective effects on ICH models [6], [7].

Brain edema is a kind of important pathophysiological change after stoke. Similarly, previous researches on VEGF and brain edema mainly focused on cerebral ischemic models, but the relationship between them was complicated and still controversial. It was demonstrated that VEGF increased blood-brain barrier (BBB) permeability and worsened brain edema [8]. However, there is evidence showing that VEGF did not aggravate brain edema [9], but rather reduce it [10]. Nevertheless, although brain edema following ICH is more serious than cerebral ischemia and often leads to poor prognosis, there has been no related research on how VEGF acts on brain edema after ICH.

Aquaporin-4 (AQP4), as the most abundant water channel in the central nervous system (CNS), plays an important role in the formation and resolution of brain edema, but has opposite effects on different brain edema types. It may be involved in formation of cytotoxic brain edema, but help to eliminate vasogenic brain edema [11]. While both of the two types are involved in brain edema following ICH [12]. Previous work from our group indicated that compared with wild type mice, AQP4 knockout aggravated brain edema, worsened neurological deficits and increased cell injury after ICH [13].

It was reported that VEGF was co-localized with AQP4 on astrocyte processes after cerebral hypoxia and BBB disruption [14]. Meanwhile, intracerebral VEGF injection highly up-regulated AQP4 mRNA and protein in the perivascular space and glia limitans externa [15]. Based on the evidences that VEGF is closely related with AOP4, as well as both of them are essential to brain edema, we speculate that the effect of VEGF on brain edema following ICH may result from regulating AQP4 expression.

Mitogen-activated protein kinase (MAPK) pathways include three main members: extracellular signal-regulated kinase (ERK), C-Jun amino-terminal kinase (JNK) and p38-MAPK, among which crosstalk often occurs. It was shown that VEGF and its receptors elicited their biological effects mainly rely on activation of phosphatidylinositol 3′-kinase (PI3K)/Akt and ERK pathways [16], [17]. In addition, the inhibitors of MAPKs suppressed AQP4 up-regulation induced by manganese-treated or oxygen-glucose deprivation and recovery [18], [19]. According to the above findings, we suppose that VEGF may regulate AQP4 expression by activating MAPK pathways.

To test these hypotheses, we injected VEGF intracerebroventricularly after ICH and examined the changes of brain edema and AQP4 expression in wild type (AQP4^+/+^) mice. We also investigated whether VEGF’s effects on brain edema are AQP4 dependent using AQP4 knock-out (AQP4^−/−^) mice. Furthermore, we studied the possible signal transduction pathways activated by VEGF to regulate AQP4 expression through astrocyte cultures.

## Materials and Methods

### Animals and Experimental Groups

Male AQP4^+/+^ and AQP4^−/−^ mice, 3–4 month-old, weighing 25–33 g, were kindly provided by Doctor Hu, Jiangsu Key Laboratory of Neurodegeneration, Department of Anatomy, Histology and Pharmacology of Nanjing Medical University in China. Mice were kept in the environment of a 12 h light/dark cycle with free access to food and water. Our animal studies and protocol was approved by the Institutional Animal Care and Use Committee of Fudan University.

AQP4^+/+^ and AQP4^−/−^ mice were randomly divided into five groups: Group 1: Control. Group 2: Control plus VEGF. Group 3: Sham operation. Group 4: ICH. Group 5: ICH plus VEGF. The parameters were detected 1 d, 3 d and 7 d after drugs injection and each parameter of each group contained six mice.

### Mouse ICH Model and Drugs Injection

Mice were anesthetized with 10% chloralhydrate (350 mg/kg) and were placed in a stereotaxic frame (Alcott Biotech, Shanghai, China). Through a hole drilled in the skull, a 32-gauge needle was implanted into the striatum, 2.0 mm lateral to the midline, 1.0 mm anterior to the coronal suture and at a depth of 4.0 mm from the surface of the brain. Each mouse was microinjected with 5 µl of autologous whole blood (right striatum) taken from the tail vein over 10 min using a 5 µl microinfusion pump (ALC-IP600, Alcott Biotech). Then the needle was pulled out without blood reflux after 5 min dwelling and the wound was sutured. Only the mouse observed neurological deficit was regarded as a successful model. The mice in sham operation group had the same operation but no blood was injected. Recombinant human VEGF165 (rhVEGF165, Millipore, Billerica, MA, USA) was injected intracerebroventricularly (0.9 mm lateral to the midline, 0.1 mm posterior to the coronal suture and at a depth of 3.1 mm from the surface of the brain) 1 d after ICH with a dose of 3 µg/kg according to previous investigations [10], [20] and our preliminary experiment. The same dose of phosphate buffer solution (PBS) was injected intracerebroventricularly as control.

### Astrocyte Culture and Groups

Astrocyte culture was prepared as previously reported [21]. Cerebral cortices from neonatal Sprague-Dawley rat brains (P1–2) were removed and carefully dissected. The tissue was dissociated in DMEM containing 0.0025% trypsin/EDTA (Gibco) and passed through a 70 mm pore nylon mesh. After centrifugation, the cell pellet was resuspended in DMEM/F12 medium containing 10% fetal bovine serum (FBS), 50 U/ml penicillin, and 50 mg/ml streptomycin (all from Gibco). The cells were then plated onto 25 ml tissue culture flasks. The medium was renewed every 2 to 3 days. After incubation for 5 to 7 days, the cells were trypsinized and subcultured in 60-mm diameter culture dishes. The cell population consisted of over 95% astrocytes as determined by immunocytochemical staining with anti-glial fibrillary acidic protein (GFAP) antibody (Millipore).

The astrocytes were randomly divided into six groups. Group 1: Normal astrocytes. Group 2: VEGF(50 ng/ml). Group 3: VEGF plus SP600125 (20 µM, Invivogen, San Diego, CA, USA), a kind of JNK inhibitor. Group 4: VEGF plus U0126 (10 µM, Invivogen), a kind of ERK inhibitor. Group 5: VEGF plus SB239063 (10 µM, Enzo Life Sciences Inc., NY, USA), a kind of p38-MAPK inhibitor. Group 6: VEGF plus Ly294002 (30 µM, Invivogen), a kind of PI3K inhibitor. The doses of drugs were according to previous reports [22]–[25].

### Neurological Testing

A standardized battery of behavioral tests was used to quantify sensorimotor neurological function at 1 d, 3 d and 7 d after drug injection. The battery consisted of two tests: the postural reflex test, which examines upper-body posture while the animal was suspended by the tail, and the forelimb placing test, which examines sensorimotor integration in forelimb placing responses to visual, tactile, and proprioceptive stimuli. Neurological function was graded on a scale of 0 to 12 (normal score 0, maximal score 12) as previously described [26]. Tests were conducted by an observer blinded to the treatment group.

### Brain Water Content

The water content of the brain was measured to evaluate the formation of perihemotomal edema at 1 d, 3 d and 7 d after drug injection. Brain was rapidly removed from the skull and the olfactory bulbs and brain stem with the cerebellum were eliminated before water contents were measured, which were performed less than 2 min after the decapitation. The brain was cut into a 2 mm slice with the center as needle entry point. Six tissue samples surrounding hemotoma were removed from the slice with Pasteur pipettes of 3 mm diameter. After the wet weight was measured, the tissue was then dried at 100°C for 24 h, and its dry weight was measured. Brain water content (as a percentage) = (wet weight-dry weight)/wet weight×100%. [27].

### Brain Specific Gravity

For determination of specific gravity, a linear kerosine/bromben-zene gradient was prepared. Appropriate volumes of two stock solutions with a specific gravity of SG = 0.9750 g/cm^3^ and SG = 1.0650 g/cm^3^ were mixed with a gradient mixer (LKB Ultrograd 11300). The outflow of the mixer was connected to a graded cylinder containing a volume of 100 ml. During the filling process the cylinder was lowered to maintain a steady flow to the surface of the gradient. The cylinder, the connecting tubes, and the beakers containing the stock solutions were kept at a constant temperature of 25°C in a heated water bath. After 12 h the gradient was calibrated with 5 µ1 droplets of potassium sulfate solutions with specific calculated gravities of 1.0250 g/cm^3^, 1.0300 g/cm^3^, 1.0400 g/cm^3^, and 1.0450 g/cm^3^, respectively. The method for brain tissue samples obtaining was similar to the “brain water content” section. The samples were immersed into the gradient, and the specific gravity was read from the column exactly 90 s later [13].

### Determination of BBB Permeability with the Use of Evans Blue

Evans blue (EB, Sigma-Aldrich) in normal saline (2%, 4 ml/kg) was injected intravenously at 1 d, 3 d and 7 d after drug injection. The mice were anesthetized with 10% chloralhydrate (350 mg/kg) 1 h later and perfused with 200 ml of normal saline solution through the left cardiac ventricle. The mice were decapitated and the tissue samples surrounding hemotoma were obtained as mentioned above. The samples were homogenized in methylformamide (1 ml/100 mg brain tissue), incubated for 24 h at 60°C, and centrifuged for 5 min at 1000 rpm. The absorbance (*A*) of supernatants was analyzed at 632 nm by spectrophotometry. The amount of Evans blue (µg/g) was calculated through standard curve established by known concentrations of Evans blue [28].

### Western Blotting

Protein extraction: (1) *In vivo* experiment: AQP4^+/+^ mice was decapitated 1 d, 3 d and 7 d after drug injection and the brain hemispheres (hemorrhage) were homogenized in RIPA lysis buffer (Beyotime, Suzhou, Jiangsu, China) and 1 mM phenylmethanesulfonyl fluoride (PMSF). The homogenate was centrifuged at 12000 rpm for 15 min at 4°C, and the supernatant was used for analysis. (2) *In vitro* experiment: The culture fluid of each group of astrocytes was discarded 48 h after drugs administration and the cells were treated with RIPA lysis buffer and PMSF. Additionally, astrocytes of Group 2 were treated in the same way 0 h, 12 h, 24 h, 48 h, 72 h after drugs administration. The lysate was centrifuged at 12000 rpm for 15 min at 4°C, and the supernatant was used for analysis. The concentration of protein was analyzed by BicinChoninic Acid (BCA) protein assay kit (Beyotime). Proteins (50 µg) were loaded onto 4% stacking/12% separating SDS-polyacrylamide gels for electrophoresis, and then transferred onto nitrocellulose transfer membranes. Membranes were blocked at room temperature for 1 h with blocking solution (5% skimmed milk in Tris-buffered solution plus Tween-20 (TBST, 50 mM Tris–HCl, 150 mM NaCl, pH = 7.5, 0.1% v/v Tween-20). Membranes were then incubated overnight at 4°C with anti-AQP4 (1∶1000, Sigma-Aldrich), anti-p-JNK (1∶1000, CST, Boston, MA, USA), anti-p-ERK (1∶1000, CST), anti-p-p38 (1∶1000, CST), anti-p-Akt (1∶1000, CST) rabbit polyclonal antibody. After three 10 min washing in TBST, membranes were incubated for 1 h at room temperature with horseradish peroxidase (HRP) labeled goat anti-rabbit secondary antibody (1∶4000, Vector, Burlingame, CA, USA). The membranes were placed into ECL solution for 5 min, and then exposed. The intensity of blots was quantified using the Leica Image Processing and Analysis System. β-Actin was used as an internal control.

### Immunofluorescence

Specimen preparation: (1) *In vivo* experiment: AQP4^+/+^ mice were anesthetized with 10% chloralhydrate (350 mg/kg) 1 d, 3 d and 7 d after drug injection and perfused with normal saline solution and 4% ice-cold paraformaldehyde through the left cardiac ventricle. The brain tissues were removed and gradient dehydrated. Serial coronal sections (10 µm) were then cut on a freezing microtome (CM 1900, Leica, Wetzlar, Germany). (2) *In vitro* experiment: The coverslips full of astrocytes were taken out 48 h after drugs administration. Each group of astrocytes was fixed by 4% paraformaldehyde for 30 min. After washed in 0.01 M PBS and 0.3% Triton X-100, the sections were blocked by 10% goat serum. Sections were incubated overnight at 4°C with anti-AQP4 rabbit polyclonal antibody (1∶500, Sigma-Aldrich), and then incubated 1 h at 37°C with fluorescein isothiocyanate (FITC) coupled secondary goat anti-rabbit IgG (1∶500, Invitrogen). The astrocytes were also stained with 4′, 6-diamidino-2-phenylindole (DAPI) for highlighting the nuclei. Controls were performed by omitting primary antibodies. All controls gave negative results with no detectable labeling.

### Nissl’s Staining and Cell Counting

Brain frozen sections were obtained by method in “immunofluorescence” section. Sections were stained with toluidine blue (Sigma-Aldrich) for 20 min. After washed by distilled water, sections were separated color with 95% ethanol for 30 s. For determining cell counting, brain sections through the needle entry site starting with the first appearance of Nissl’s staining cells, extending to the most caudal parts of the hematoma zone were Nissl’s staining at 1 d, 3 d, and 7 d after drug injection (n = 6 per group). Six brain sections per mouse were selected according to the principle that it is most variability in Nissl’s staining when compared to control group. Mean number of positive cells per section was recorded [13].

### Statistical Analysis

All data were presented as mean±SEM and analyzed with SPSS12.0. Differences between groups were compared by using one-way analysis of variance (ANOVA). When the ANOVA identified significant between-group differences, Tukey’s Honestly Significant Difference (HSD) tests were used for intergroup comparisons. Two-tailed Student *t* tests were for comparisons between AQP4^+/+^ and AQP4^−/−^ mice in each group. In addition, we added two-way ANOVA with replication in “neurological testing” and “Nissl’s staining” sections to examine whether interactions existed between VEGF injection and presence of AQP4 gene.

## Results

### Expression of AQP4 Protein in each Group of AQP4^+/+^ Mice

**Figure 1:**
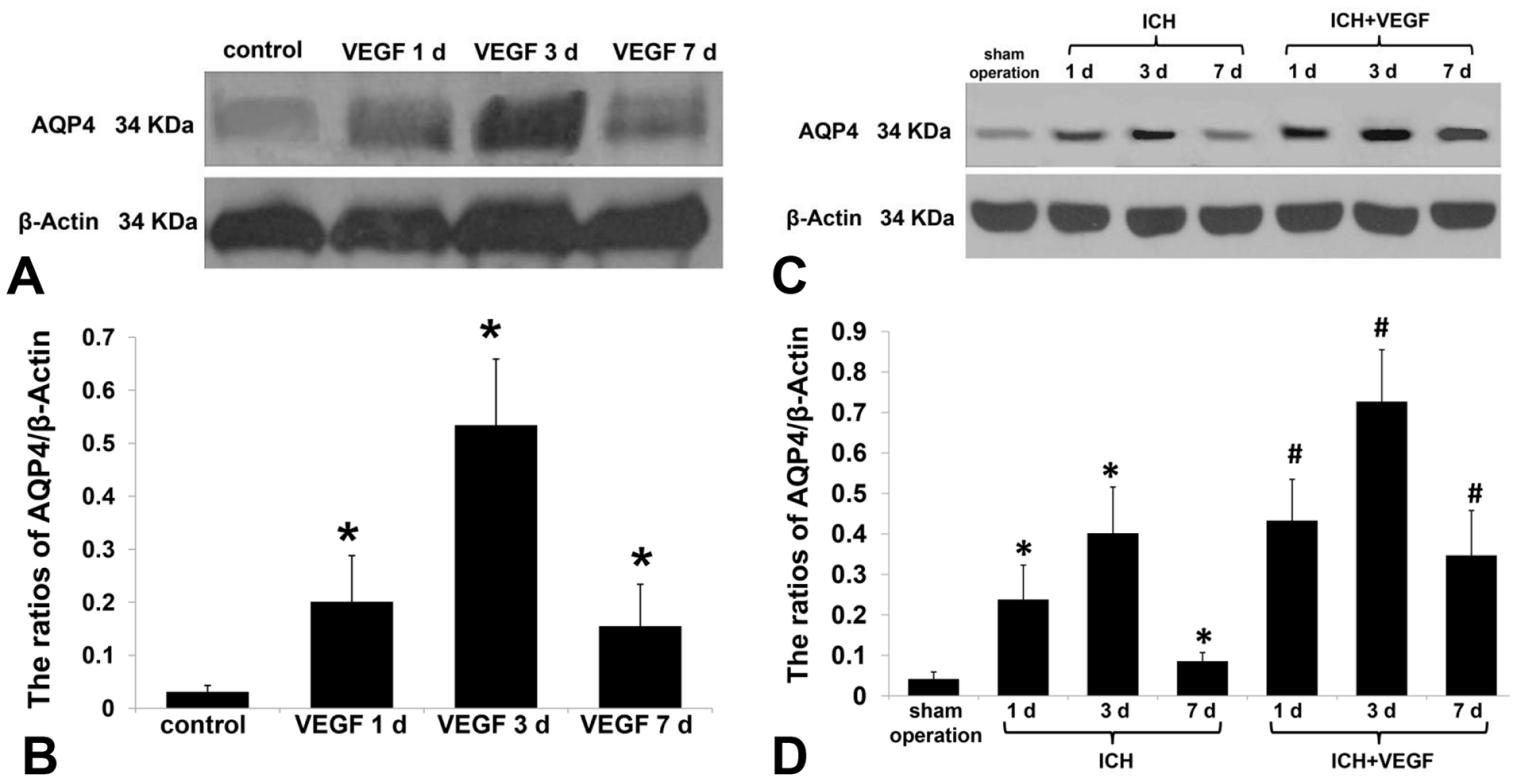
Western blot analysis of AQP4 protein expression in each normal AQP4^+/+^ group. (A, B) Western blot analysis showed rhVEGF165 injected intracerebroventricularly up-regulated AQP4 protein expression at striatum in normal AQP4^+/+^ mice at 1 d, 3 d, and 7 d (n = 6, *p<0.05 vs. control). (C, D) Western blot analysis showed rhVEGF165 up-regulated perihemotomal AQP4 protein expression at 1 d, 3 d, and 7 d after intracerebroventricular injection (n = 6, *p<0.05 vs. sham operation, #*p*<0.05 vs. ICH).

**Figure 2:**
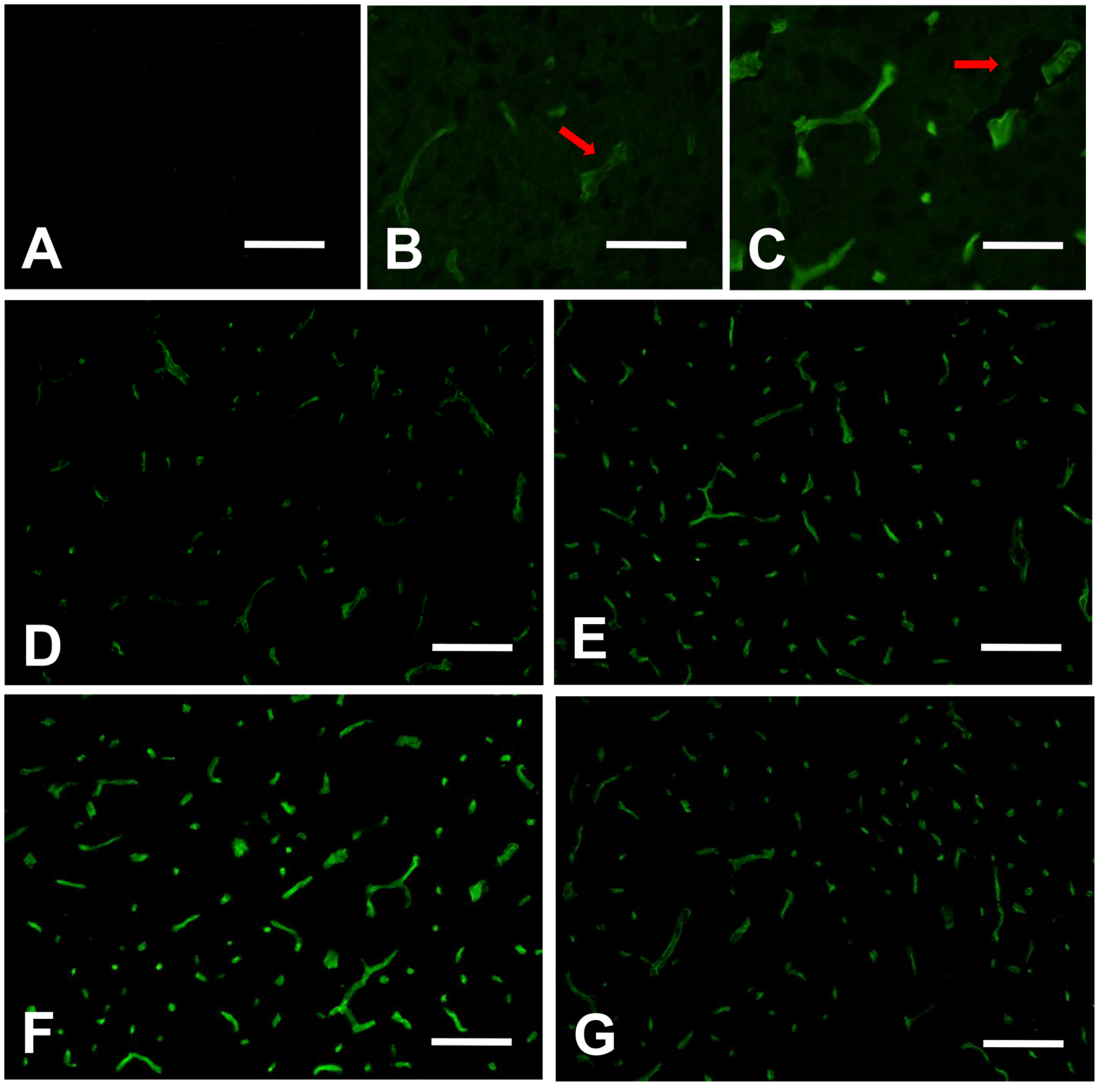
Immunofluorescence of AQP4 protein expression in each normal AQP4^+/+^ group. (A) Negative control without primary antibody gave negative result with no detectable AQP4 labeling. (B, C) High magnification for groups of control and VEGF 3 d after injection showed AQP4 labeling was concentrated in glial end-feet surrounding intracerebral capillaries (arrows). (D–G) Groups of control, VEGF 1 d, 3 d, and 7 d after injection. Vermiform AQP4 with green fluorescence was abundantly expressed after rhVEGF165 injection. Immunofluorescence revealed similar results as Western blot analysis. Scale bar: A, D–G: 100 µm; B, C: 50 µm.

**Figure 3:**
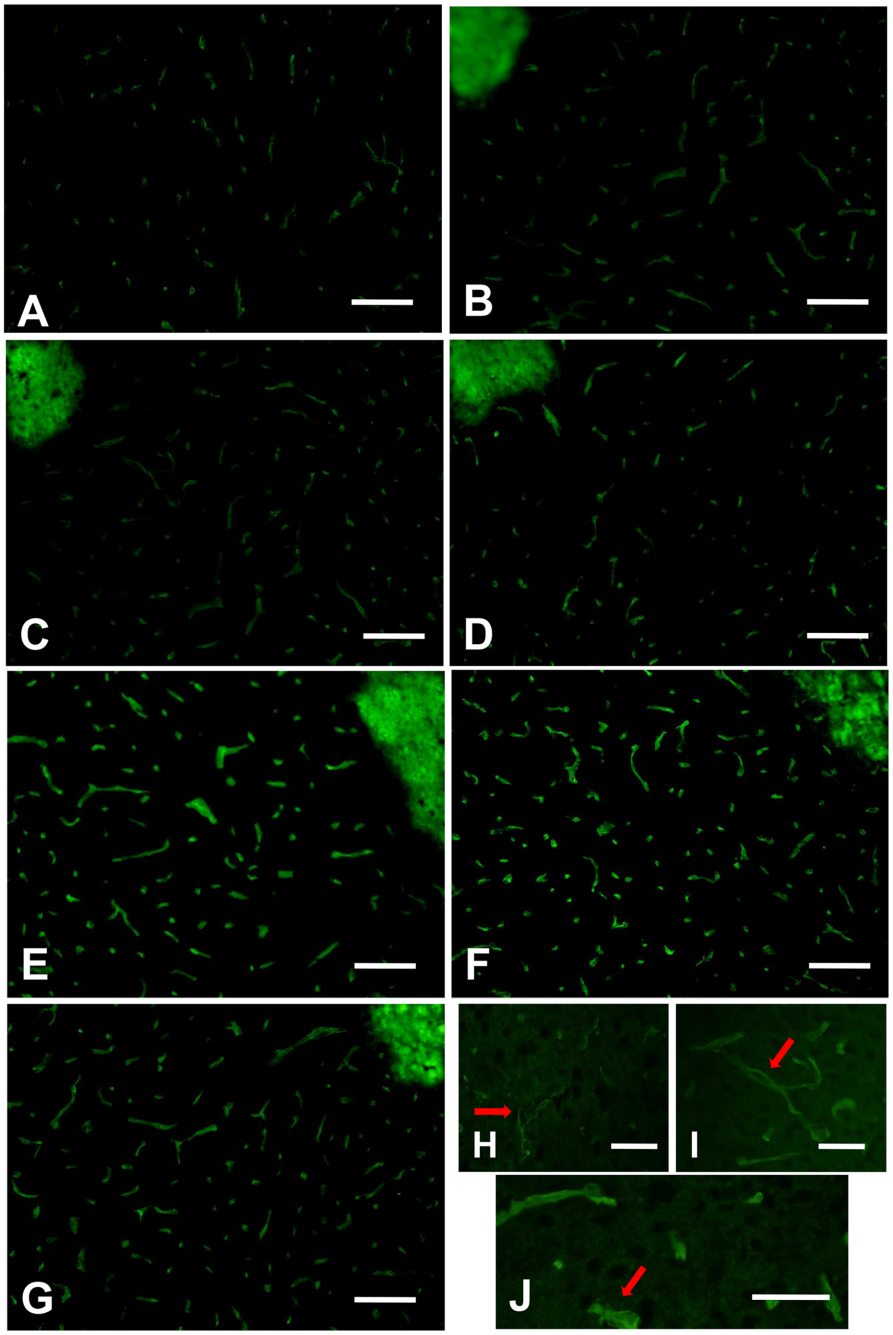
Immunofluorescence of AQP4 protein expression in each ICH AQP4^+/+^ group. (A) Group of sham operation. (B–D) Groups of ICH 1 d, 3 d, and 7 d. (E–G) Groups of ICH plus VEGF 1 d, 3 d, and 7 d after injection. (H–J) High magnification for groups of sham operation, ICH and ICH plus VEGF 3 d after injection. Arrows show AQP4 labeling is concentrated in glial end-feet surrounding intracerebral capillaries. Scale bar: A–G: 100 µm; H–J: 50 µm.

Immunofluorescence showed vermiform AQP4 protein with green fluorescence abundantly expressed in groups of VEGF, ICH and ICH plus VEGF, all peaked at 3 d after drug injection. High magnification showed AQP4 labeling was concentrated in glial end-feet surrounding intracerebral capillaries (arrows) as described previously [29], [12]. Meanwhile, both Western blot and immunofluorescence revealed an increase of AQP4 protein in striatum at 1 d, 3 d, and 7 d after rhVEGF165 injected intracerebroventricularly in normal mice (*p*<0.05). Furthermore, perihemotomal AQP4 protein expression was increased by intracerebroventricular injection of rhVEGF165 at the three time points after ICH. The Statistical differences were found in semi-quantitive analysis of Western blot (*p*<0.05) (Figure 1–3).

### Brain Water Content of AQP4^+/+^ and AQP4^−/−^ Mice

**Figure 4:**
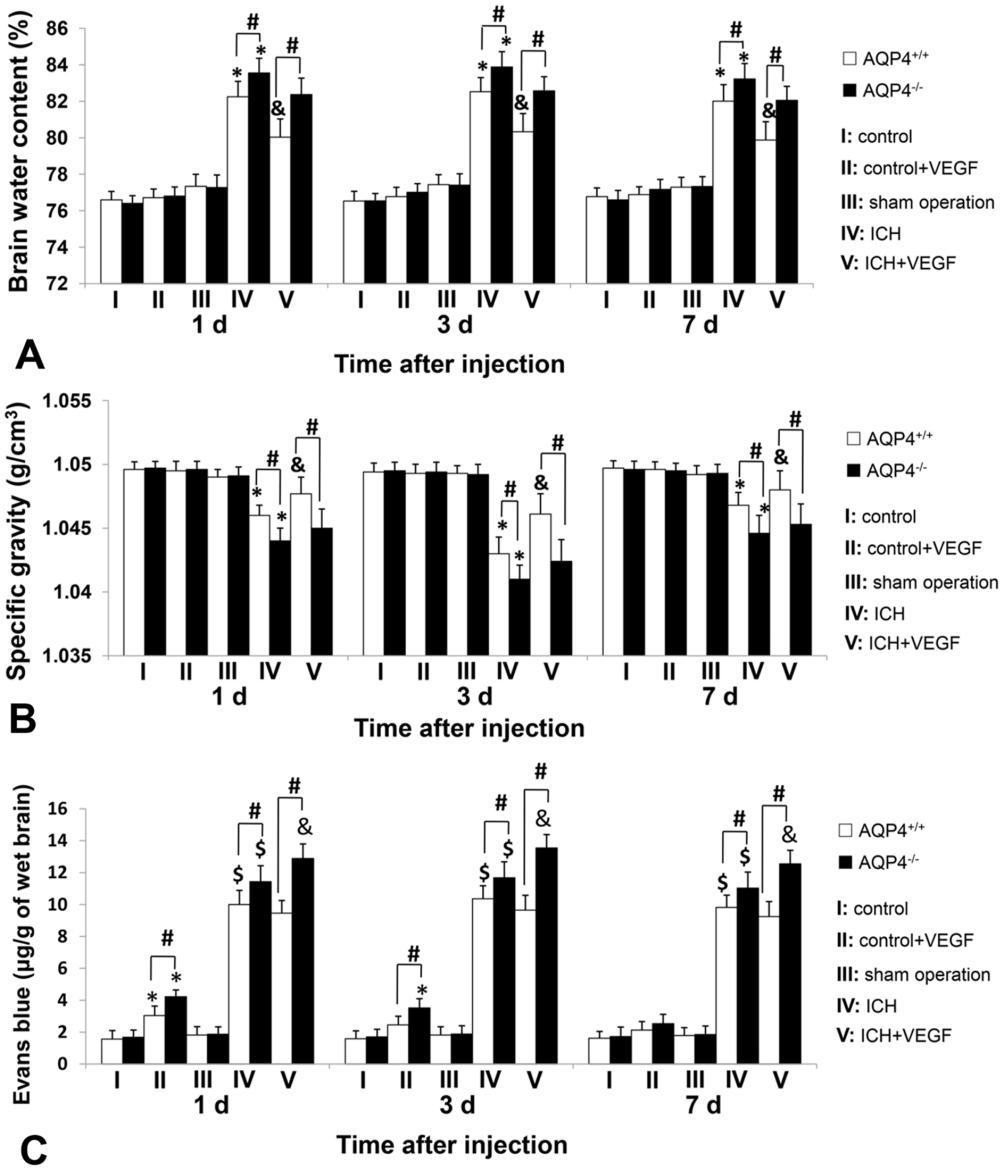
Brain water content, brain specific gravity and EB extravasation amount of AQP4^+/+^ and AQP4^−/−^ mice. (A) Brain tissue water content surrounding the hemotoma in AQP4^+/+^ mice was markedly reduced by rhVEGF165 at 1 d, 3 d, and 7 d after intracerebroventricular injection (n = 6, *p<0.05 vs. sham operation; & *p*<0.05 vs. ICH), but no effect was observed in AQP4^−/−^ mice. AQP4^−/−^ mice had more perihemotomal brain tissue water content than AQP4^+/+^ mice in both ICH and ICH plus VEGF groups (n = 6, #*p*<0.05). (B) The specific gravity of brain tissue surrounding hematoma from AQP4^+/+^ mice was significantly decreased by rhVEGF165 at 1 d, 3 d, and 7 d after injected intracerebroventricularly (n = 6, *p<0.05 vs. sham operation; & *p*<0.05 vs. ICH), while no influence was detected in AQP4^−/−^ mice. In addition, AQP4^+/+^ mice had more specific gravity of perihemotomal brain tissues than AQP4^−/−^ mice in both ICH and ICH plus VEGF groups (n = 6, #*p*<0.05). Put together, results of the two sections were consistent with each other. (C) In normal mice, the amount of EB extravasation was increased 1 d after intracerebroventricular rhVEGF165 injection in both types of mice, but this effect was only detected in AQP4^−/−^ mice at 3 d (n = 6, *p<0.05 vs. control). No change was found at 7 d. RhVEGF165 had no effect on EB extravasation resulting from ICH whether 1 d, 3 d, or 7 d after intracerebroventricular injection in AQP4^+/+^ mice, while an increasing effect in AQP4^−/−^ mice was found at all time points observed (n = 6, $ *p*<0.05 vs. sham operation; & *p*<0.05 vs. ICH). Meanwhile, this section also showed that AQP4^−/−^ mice had more EB extravasation than AQP4^+/+^ mice in VEGF 1 d, 3 d, all time points of ICH and ICH plus VEGF groups (n = 6, #*p*<0.05).

There was no change of brain water content in normal AQP4^+/+^ and AQP4^−/−^ mice after intracerebroventricular injection of rhVEGF165. AQP4^−/−^ mice had more severe brain edema after ICH than AQP4^+/+^ mice at each time point (*p*<0.05). Injection of rhVEGF165 intracerebroventricularly after ICH alleviated brain edema surrounding hemotoma in AQP4^+/+^ mice at 1 d (80.03%±0.78% vs. 82.25%±0.84%), 3 d (80.33%±0.79% vs. 82.53%±0.77%) and 7 d (79.88%±0.74% vs. 82.01%±0.90%) (*p*<0.05), but had no effect on AQP4^−/−^ mice (Figure 4A).

### Brain Specific Gravity of AQP4^+/+^ and AQP4^−/−^ Mice

It is another method for measuring perihemotomal brain edema, and the main advantage of specific gravity measurements for evaluation of brain edema is the possibility to obtain reliable results in small brain tissues. Expectably, the results were in accord with brain tissues water content. Compared to AQP4^+/+^ mice, AQP4 deletion significantly decreased specific gravity of brain tissue surrounding hematoma at the three time points after ICH (*p*<0.05). The increase of brain specific gravity was observed in AQP4^+/+^ mice 1 d, 3 d, and 7 d after rhVEGF165 intracerebroventricular injection (*p*<0.05), but not AQP4^−/−^ mice (Figure 4B).

### EB Extravasation Amount of AQP4^+/+^ and AQP4^−/−^ Mice

Since we focused on perihemotomal brain edema, we chose brain tissues also surrounding hemotoma for investigating VEGF’s effect on BBB permeability. In normal mice, the amount of EB extravasation was increased 1 d after intracerebroventricular rhVEGF165 injection in both types of mice and it was more in AQP4^−/−^ mice (*p*<0.05). But at 3 d, increase was only detected in AQP4^−/−^ mice (*p*<0.05). The amount of EB extravasation was returned to baseline at 7 d. We also found AQP4 deletion increased EB extravasation after ICH (*p*<0.05). Furthermore, injection of rhVEGF165 intracerebroventricularly after ICH had no change of EB extravasation in AQP4^+/+^ mice at the chosen time points, but developed an increasing effect in AQP4^−/−^ mice (*p*<0.05) (Figure 4C).

### Neurological Scores of AQP4^+/+^ and AQP4^−/−^ Mice

**Figure 5:**
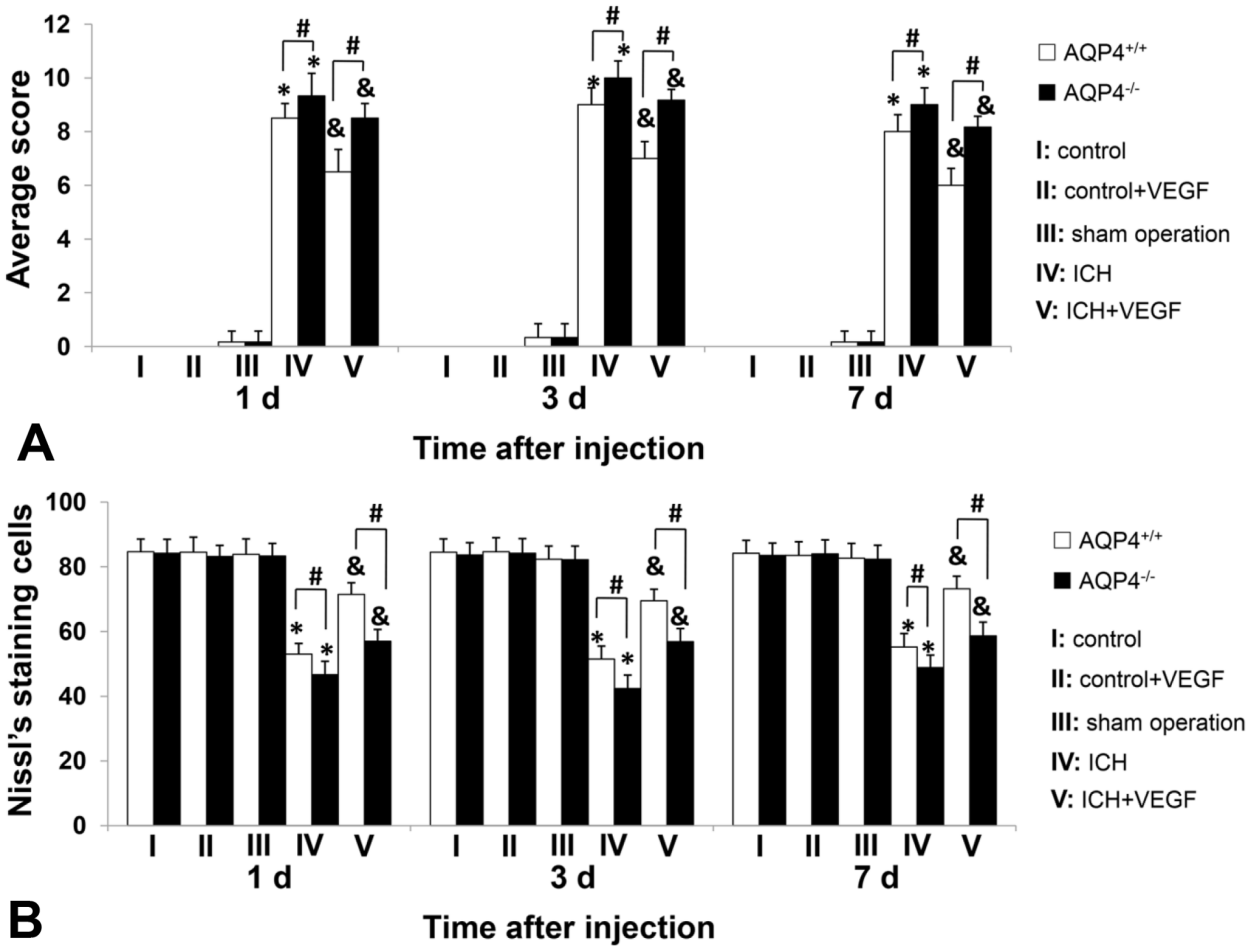
Neurological scores and Nissl’s staining cell counting of AQP4^+/+^ and AQP4^−/−^ mice. (A) RhVEGF165 alleviated neurological deficits due to ICH of AQP4^+/+^ and AQP4^−/−^ mice at 1 d, 3 d, and 7 d after injected intracerebroventricularly (n = 6, *p<0.05 vs. sham operation; & *p*<0.05 vs. ICH). Moreover, neurological deficits were more severe in AQP4^−/−^ mice than AQP4^+/+^ mice after ICH and rhVEGF165 injection (n = 6, #*p*<0.05). Interactions existed between VEGF administration and presence of AQP4 gene (*p* = 0.033, 0.024 and 0.024 of 1 d, 3 d and 7 d). (B) Nissl’s staining cells surrounding hemotoma in both AQP4^+/+^ and AQP4^−/−^ mice were significantly increased by rhVEGF165 1 d, 3 d, and 7 d after intracerebroventricular injection (n = 6, *p<0.05 vs. sham operation; & *p*<0.05 vs. ICH). Meanwhile, more Nissl’s staining cells were found perihemotoma in AQP4^+/+^ mice than AQP4^−/−^ mice at any observed time points in both ICH and ICH plus VEGF groups (n = 6, #*p*<0.05). Interactions existed between VEGF administration and presence of AQP4 gene (*p* = 0.013, 0.031 and 0.023 of 1 d, 3 d and 7 d).

There was no influence of rhVEGF165 injected in normal state on neurological functions of whether AQP4^+/+^ or AQP4^−/−^ mice. The neurological functions were significantly worsened after ICH with the peak at 3 d (*p*<0.05), which matched the degree of brain edema in this study. Meanwhile, more severe in AQP4^−/−^ mice than AQP4^+/+^ mice at each time point after ICH (*p*<0.05). Injection of rhVEGF165 intracerebroventricularly alleviated neurological deficits of both types of mice (*p*<0.05), while the neurological functions were still better in AQP4^+/+^ mice (*p*<0.05). To investigate whether this effect of VEGF had something to do with AQP4, we studied the interactions between VEGF injection and presence of AQP4 gene by two-way ANOVA with replication. The *F* values of 1 d, 3 d and 7 d were 5.21, 5.98 and 5.98 with the corresponding *p* values of 0.033, 0.024 and 0.024, suggesting presence of interactions (Figure 5A).

### Nissl’s Staining and Cell Counting of AQP4^+/+^ and AQP4^−/−^ Mice

**Figure 6:**
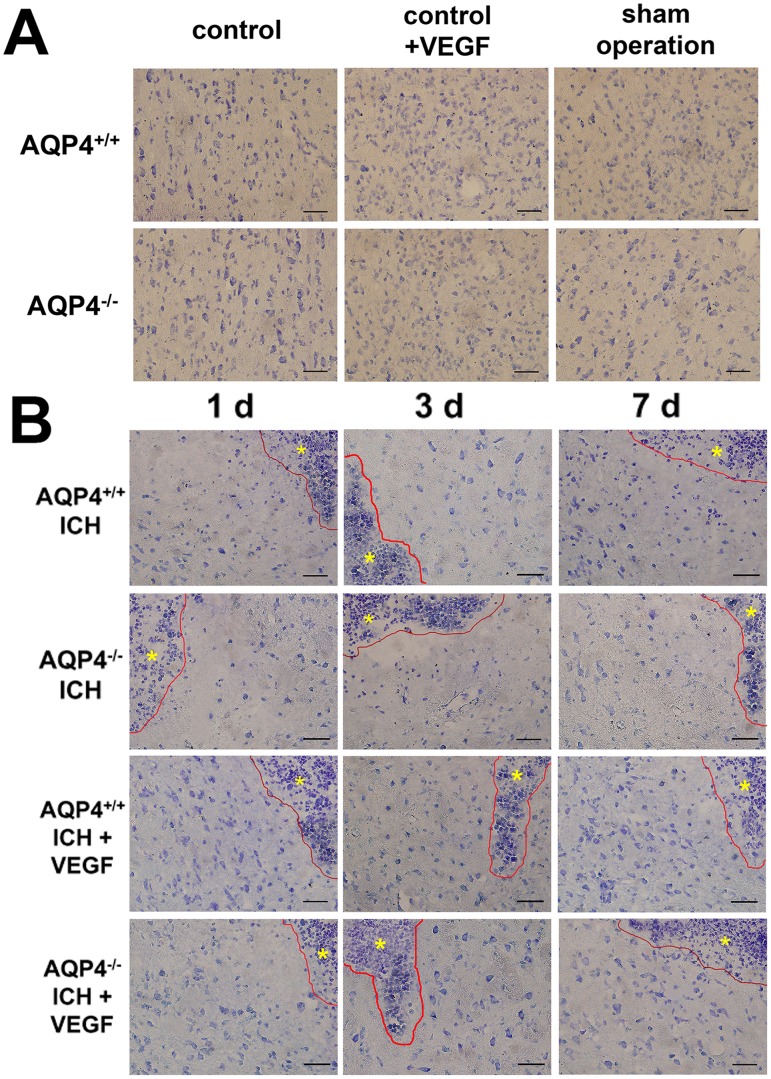
Nissl’s staining of AQP4^+/+^ and AQP4^−/−^ mice. Injection of rhVEGF165 intracerebroventricularly had no effect on Nissl’s staining in normal mice. At the three time points, marked neuron loss perihemotoma were found in AQP4^−/−^ mice when compared to AQP4^+/+^ mice. Besides, rhVEGF165 increased perihemotomal Nissl’s staining cells in both AQP4^+/+^ and AQP4^−/−^ mice. (A) Groups of control, control plus VEGF and sham operation at 3 d. (B) Groups of ICH and ICH plus VEGF at 1 d, 3 d and 7 d. Astericks represent the hemotoma zones. Scale bar: 50 µm.

Nissl’s staining was used for evaluating neuronal death resulting from ICH and Nissl’s staining positive cells were survival neurons. The changing trend of Nissl’s staining cells can be found in microscopic images (Figure 6). We made quantitive analysis by means of positive cell counting. RhVEGF165 intracerebroventricular injection had no influence on neuronal death in both types of normal mice. There were less Nissl’s staining cells found perihemotoma after ICH in AQP4^−/−^ mice than AQP4^+/+^ mice (*p*<0.05). RhVEGF165 injected intracerebroventricularly increased Nissl’s staining cells in both types of mice after ICH at the time points (*p*<0.05), but the above difference between them still existed (*p*<0.05). Similarly, two-way ANOVA with replication showed *F* values of 1 d, 3 d and 7 d were 7.44, 5.39 and 6.07 with the corresponding *p* values of 0.013, 0.031 and 0.023, suggesting interactions between VEGF administration and presence of AQP4 gene existed (Figure 5B).

### Expression of Phosphorylation of MAPKs and Akt by VEGF at Different Time Course

**Figure 7:**
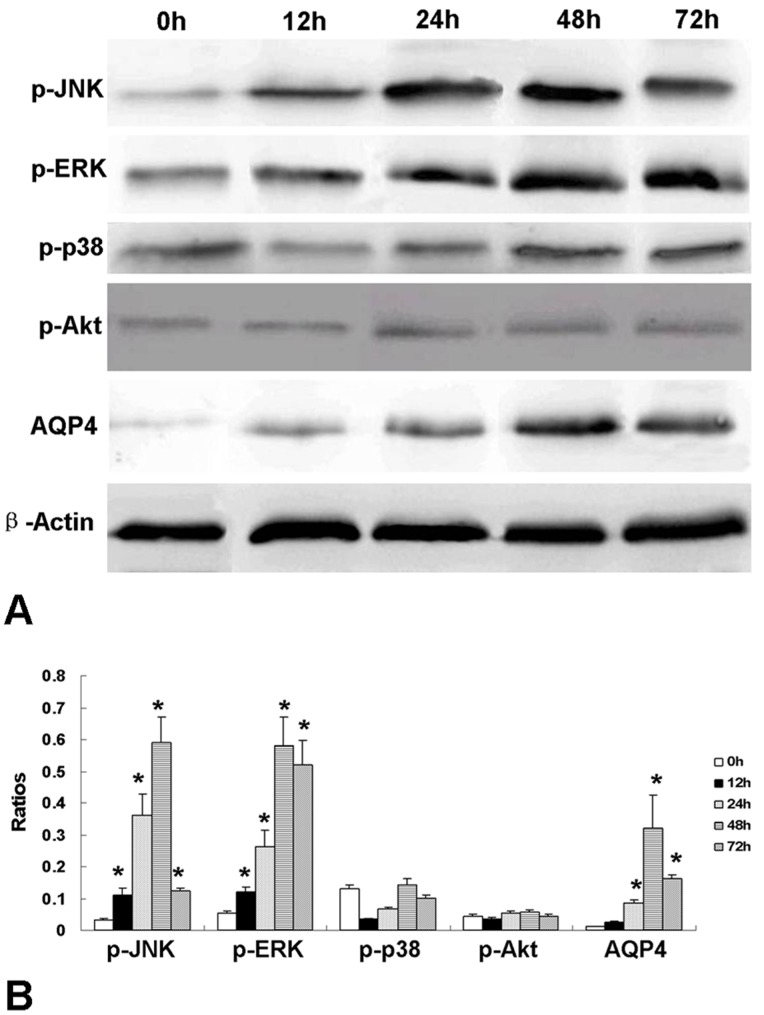
Western blot of phosphorylation of MAPKs and Akt influenced by rhVEGF165. RhVEGF165 up-regulated p-JNK and p-ERK at 12 h after administration and expression of the two proteins reached maximum at 48 h (n = 6, *p<0.05 vs. 0 h). While no up-regulation of p-p38 or p-Akt was observed within 72 h after rhVEGF165 administration. Besides, expression of AQP4 protein was increased by rhVEGF165 after 24 h and peaked at 48 h (n = 6, *p<0.05 vs. 0 h). (A) Western blot image. (B) Semi-quantification analysis.

Western blot including semi-quantitive analysis showed that p-JNK and p-ERK were up-regulated 12 h after rhVEGF165 administration and peaked at 48 h (*p*<0.05). While no up-regulation of p-p38 or p-Akt was observed within 72 h after rhVEGF165 administration. Meanwhile, expression of AQP4 protein was increased by rhVEGF165 after 24 h and peaked at 48 h. Therefore, up-regulation of AQP4 protein occurred 12 h later than p-JNK and p-ERK, but they all reached maximum at 48 h after rhVEGF165 administration (Figure 7).

### Expression of AQP4 Protein in each Group of Cultured Astrocytes

**Figure 8:**
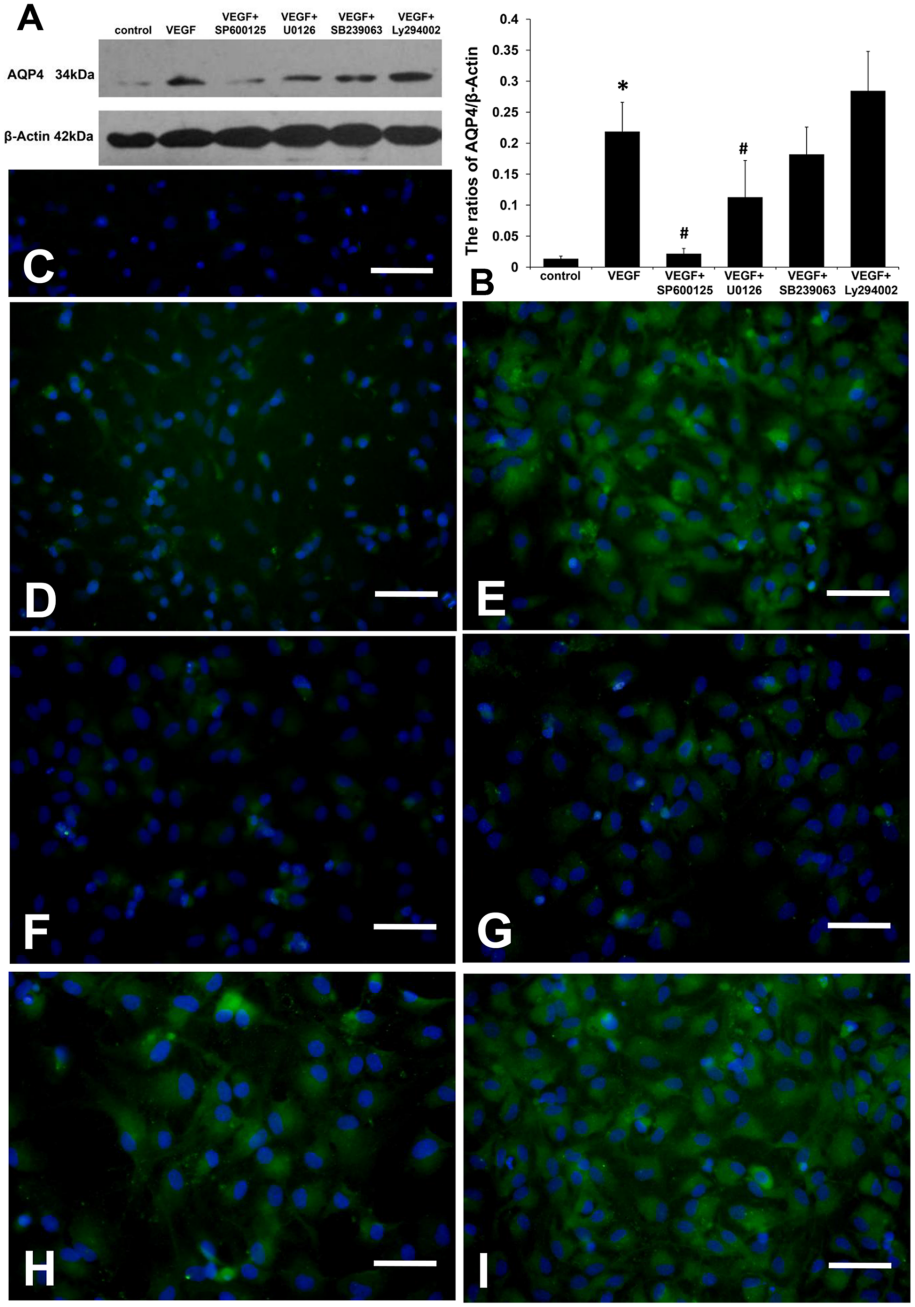
AQP4 protein expression in each astrocyte group. (A) The Western blot image of AQP4 protein. (B) Semi-quantification showed that rhVEGF165 increased AQP4 protein expression in astrocytes, which was inhibited by SP600125 and U0126, but not SB239063 or Ly294002 (n = 6, *p<0.05 vs. control, #*p*<0.05 vs. VEGF). (C) Negative control gave negative result without detectable AQP4 labeling. (D) Group of control. (E) Group of VEGF. (F) Group of VEGF plus SP600125. (G) Group of VEGF plus U0126. (H) Group of VEGF plus SB239063. (I) Group of VEGF plus Ly294002. Nuclei are highlighted by DAPI staining. Scale bar: 50 µm.

Since AQP4 protein, p-JNK and p-ERK all peaked at 48 h after rhVEGF165 administration, we chose this time point in this section. To present the morphology of AQP4 protein in cultured astrocytes, we highlighted the nuclei by DAPI staining. Different from the polarized distribution in brain tissues, expression of AQP4 protein was found homogeneously distributed in the cytoplasm of cultured astrocytes. Both Western blot and immunofluorescence revealed that rhVEGF165 up-regulated AQP4 protein expression of astrocytes (*p*<0.05). This was inhibited by SP600125 and U0126 (*p*<0.05), but not SB239063 or Ly294002 (Figure 8).

## Discussion

In this study, we investigated the effects of VEGF on brain edema following ICH for the first time, and tested whether it was AQP4 dependent. VEGF is closely related with AQP4. They were detected to be co-expressed on astrocyte processes after cerebral hypoxia and BBB disruption as well as in meningioma by double immunofluorescence labeling [14], [30]. Moreover, VEGF can up-regulate AQP4 expression in CNS under physiological and pathological states [15], [31]. In the present study, AQP4 protein was increased in striatum at 1 d after injection of rhVEGF165 intracerebroventricularly and peaked at 3 d, which was consistent with the reported change trend of AQP4 expression induced by VEGF [15]. It indicates that increase of VEGF levels in cerebro-spinal fluid (CSF) can up-regulate AQP4 expression in other regions of brain. We first investigated the effect of VEGF on AQP4 expression after ICH and found that intracerebroventricular rhVEGF165 injection significantly up-regulated perihemotomal AQP4 protein expression at the three time points. These findings provide the possibility of AQP4 being involved in exogenous VEGF’s effects on ICH.

It is still controversial how VEGF acts on brain edema. Taking cerebral ischemic models for example, different studies showed various effects of VEGF on brain edema that it can reduce, not aggravate, or worsen it [8]–[10]. These different effects may be due to the differences of drug administration times, injection ways, doses and observation time points. However, although brain edema following ICH is far more serious and dangerous to life and the strategy for the treatment is limited, no relevant researches have referred to the roles played by VEGF. In the present study, we focused on the brain edema surrounding hemotoma by measuring brain tissue water content and specific gravity. The main advantage of the latter is the possibility to obtain reliable results in small tissue samples. The two sections revealed consistent results. In addition, it was reported that late administration of VEGF provided neuroprotective effect to cerebral ischemia [5], [32], [33], and our preliminary experiment on ICH also obtained similar results. So we chose 1 d after ICH as the time point for rhVEGF165 injection. We first found perihemotomal brain edema was markedly reduced by rhVEGF165 at 1 d, 3 d, and 7 d after injected intracerebroventricularly. Since VEGF can up-regulate AQP4 expression after ICH, we further studied whether VEGF’s effect on brain edema was AQP4 dependent. We demonstrated that AQP4^−/−^ mice had more severe brain edema than AQP4^+/+^ mice and the protective effect on brain edema of intracerebroventricular rhVEGF165 injection was only observed in AQP4^+/+^ mice, but not in AQP4^−/−^ mice. Since the absence of AQP4 causes the significant difference, combined with our previous study, we speculate that AQP4 may play an important role in VEGF’s effect on brain edema after ICH.

Two main types of brain edema are classified as cytotoxic edema and vasogenic edema. By means of observation to appropriate brain edema models in AQP4 null mice versus wild types, it has been proven that AQP4 is involved in formation of cytotoxic edema and elimination of vasogenic edema [11]. It remains complicated in brain edema following ICH. As shown in animal models, injection of blood into the brain induces edema formation through the activation of thrombin, plasminogen activator and urokinase, leading to inflammatory cells activation and BBB disruption, causing vasogenic edema. This mechanism starts several hours after ICH and peaks at several days. Then substances from CNS cells disruption and red blood cells lysis result in secondary cellular injury that gives rise to cytotoxic edema. The mixed edema is sustained by these degradation products and lasts about 2 to 3 weeks. So multiple forms of edema are present after ICH, but the main form is probably vasogenic [34], [35]. Therefore, the three time points chosen in our study are just in phases of vasogenic edema formation, peak, and elimination after ICH. Based on all the findings, we suppose that the reduction of brain edema by VEGF in the three phases of ICH results from up-regulating AQP4 expression to enhance elimination of vasogenic edema. However, we did not find aggravation of brain edema by VEGF after ICH in AQP4 deletion mice. So it is possible that other mechanisms are involved in VEGF’s protective effect to brain edema following ICH, which needs further investigations.

Since the main brain edema form of ICH is vasogenic, the integrity of BBB is also very essential. We found intracerebroventricular rhVEGF165 injection increased EB extravasation in normal two types of mice at 1 d and only had this effect at 3 d in AQP4^−/−^ mice, suggesting an acute and transient disruptive effect on BBB. Similar effects of changing BBB permeability and ultrastructures were reported previously [36], [37]. The mechanisms include formation of interendothelial gaps and segmental fenestrae-like narrowings in endothelium, disruption of tight junction proteins [38], [39]. Nevertheless, the role of VEGF acting on BBB in pathological conditions is still unconfirmed. For instants, VEGF was reported to increase, not worsen or reduce BBB permeability after cerebral ischemia [40], [41]. We paid attention to BBB permeability surrounding hemotoma and first found that VEGF did not change EB extravasation in AQP4^+/+^ mice at all chosen time points (1 d, 3 d, and 7 d), indicating that VEGF has no effects on BBB permeability. So there must be some protective mechanisms involved. VEGF is considered to be a protective factor to endothelial cells, the important component of BBB in case of pathological damage [42], which may counteract with the damage effects mentioned above.

Furthermore, we also investigated AQP4 related mechanisms. Previous studies highlighted the importance of AQP4 in maintaining integrity of BBB in development and mature individuals [43], [44]. In our study, more serious BBB damage was observed in AQP4^−/−^ mice in normal state as well as after ICH, and VEGF’s disruptive effects on BBB was only found in AQP4^−/−^ mice. Meanwhile, AQP4 is highly concentrated in astrocyte foot processes surrounding capillaries, which is an important target for BBB integrity maintenance. In addition, in our previous study, electron micrographs showed that expression of AQP4 suppressed the perivascular space widening and endothelial cell swelling when compared with the AQP4^−/−^ mice group [13]. Therefore, we speculate that increase of AQP4 expression after ICH can maintain BBB integrity by protecting astrocyte and tight junction, which is also one of the positive effects of VEGF on BBB permeability after ICH.

Evidence suggests that VEGF can reduce neuronal death [22], and we first got positive effect of VEGF on protecting neuron surrounding hemotoma by Nissl’s staining and positive cell counting. The mechanisms include down-regulation of neurotoxic proteins, e.g. Aβ1–42, as well as up-regulation of anti-apoptotic and neurotrophic proteins, e.g. pigment epithelial-derived factor (PEDF) [22]. Meanwhile, VEGF is considered to increase phosphorylation of inhibitor κB-α (IκB-α) and nuclear translocation of the transcription factor κB (NFκB), contributing to the cell protective effects [45]. Moreover, we found interactions between VEGF administration and presence of AQP4 gene by two-way ANOVA with replication. It suggests that AQP4 is also involved in this effect of VEGF. It was demonstrated that AQP4 deletion increased glutamate levels in brain but down-regulated glutamate uptake and glutamate transportor1 expression in astrocytes [46], [47]. Therefore, AQP4 may regulate the level of glutamate, a neurotoxic factor, to inhibit neuronal death. Furthermore, we simultaneously found rhVEGF165 markedly improved neurological function after ICH, as defined by other studies in different pathological conditions [48], [49]. Apart from the neuropretective mechanisms mentioned above, VEGF can also be taken as an independent factor to neurological function after ICH [50]. Similarly, AQP4 was involved in this effect of VEGF according to our data. The possible reason may be explained by the effects of AQP4 on the reduction of brain edema, BBB permeability and neuronal death.

Our study also demonstrated that VEGF up-regulated AQP4 with increase of p-JNK and p-ERK in cultured astrocytes, and the inhibitors of JNK and ERK prevented AQP4 up-regulation. This implies up-regulation of AQP4 by VEGF may result from activation of JNK and ERK pathways. It has been proven that biological effects of VEGF mainly rely on activation of PI3K/Akt and ERK pathways after combination with VEGF receptor-2 [16], [17]. Meanwhile, the inhibitors of ERK and p38-MAPK were reported to suppress AQP4 up-regulation induced by manganese-treated or oxygen-glucose deprivation and recovery [18], [19]. This can explain the effect of ERK inhibitor in our *in vitro* experiment. It was reported that the phosphorylation of JNK kinases was observed in vascular endothelial cells after VEGF stimulation, and the angiogenesis effect of VEGF was inhibited by JNK inhibitor, suggesting VEGF can also activate JNK pathway [51]. Moreover, phosphorylation of JNK was persistently up-regulated in astrocyte after pathological pain and similar tendency of AQP4 expression was observed [52]. Put these findings together with the fact that cross talk exists generally among MAPK family, it is not hard to explain why JNK is involved. Although p38-MAPK was mentioned in several studies for regulating AQP4 expression, it was not involved in our study. Similar findings about activin receptor-like kinase 1 inhibiting human microvascular endothelial cell migration were also reported [53]. We speculate that different signal transduction pathways may be activated by different stimulations to regulate AQP4 expression.

In conclusion, it is the first time to demonstrate that VEGF has a protective effect on brain edema surrounding hemotoma following ICH. Besides, we also find that VEGF can reduce neurological deficits and neuronal death, but has no influence on BBB permeability after ICH. The above effects are closely related with AQP4 up-regulation through comparing between wild type and AQP4 deletion mice. In addition, our study reveals that the AQP4 up-regulation by VEGF maybe result from the activation of JNK and ERK pathways. The current study provides double targets to treatment of brain edema following ICH, which can present new insights to drugs development. Further researches may focus on if VEGF can elicit the chronic effects such as neurogenesis, angiogenesis etc. on ICH, and whether they are AQP4 dependent. Meanwhile, other mechanisms besides AQP4 of VEGF to reduce brain edema following ICH can also be another emphasis for future work.
